# Establishment of a cell senescence related prognostic model for predicting prognosis in glioblastoma

**DOI:** 10.3389/fphar.2022.1034794

**Published:** 2022-12-06

**Authors:** Hongbin Li, Zhuozhou Wang, Chengde Sun, Shuangjia Li

**Affiliations:** ^1^ Department of Neurosurgery, First Affiliated Hospital of Jiamusi University, Jiamusi, China; ^2^ Department of Cardiology, First Affiliated Hospital of Jiamusi University, Jiamusi, China; ^3^ Department of Emergency Medicine, First Affiliated Hospital of Jiamusi University, Jiamusi, China

**Keywords:** glioblastoma, cell senescence, decision trees, molecular subtypes, prognostic

## Abstract

**Background:** Glioblastoma (GBM) is highly malignant and has a worse prognosis with age, and next-generation sequencing (NGS) provides us with a huge amount of information about GBM.

**Materials and Methods:** Through the enrichment scores of cell senescence-related pathways, we constructed a consensus matrix and mined molecular subtypes and explored the differences in pathological, immune/pathway and prognostic. Also we identified key genes related to cell senescence characteristics using least absolute shrinkage and selection operator (Lasso) regression and univariate COX regression analysis models. The use of risk factor formats to construct clinical prognostic models also explored the differences in immunotherapy/chemotherapy within the senescence-related signatures score (SRS.score) subgroups. Decision trees built with machine learning to identify the main factors affecting prognosis have further improved the prognosis model and survival prediction.

**Results:** We obtained seven prognostic-related pathways related to cell senescence. We constructed four different molecular subtypes and found patients with subtype C1 had the worst prognosis. C4 had the highest proportion of patients with IDH mutations. 1005 differentially expressed genes (DEGs) were analyzed, and finally 194 Risk genes and 38 Protective genes were obtained. Eight key genes responsible for cell senescence were finally identified. The clinical prognosis model was established based on SRS.score, and the prognosis of patients with high SRS.score was worse. SRS.score and age were the vital risk factors for GBM patients through decision tree model mining.

**Conclusion:** We constructed a clinical prognosis model that could provide high prediction accuracy and survival prediction ability for adjuvant treatment of patients with GBM.

## Introduction

Glioma is the most malignant and common primary malignant brain tumor, characterized by high morbidity and mortality and a poor prognosis (Sung et al., 2021). Glioblastoma (GBM) which originates from glial stem cells or progenitor cells, can exhibit astonishing cellular heterogeneity (Gimple et al., 2019). It can be divided into primary glioblastoma and secondary glioblastoma (Ohgaki and Kleihues, 2013). Mutations in isocitrate dehydrogenase 1 (IDH1) and IDH2 drive the development of gliomas, which occur in most low-grade gliomas and secondary high-grade gliomas, and they enable the isocitrate dehydrogase (NADP+) activity (Geisbrecht and Gould, 1999; Haddock et al., 2022). More than 90% of glioblastomas are IDH wild-type tumors. Incidence increased with age and was more frequent in male (Le Rhun et al., 2019). In addition to histological variations, large-scale genetic and epigenetic analytical studies allowed the differentiation of several molecular subgroups of IDH wild-type glioblastoma, characterized by unique DNA methylation patterns associated with characteristic mutations and expression profiles (Capper et al., 2018). Therefore, it is critical to elucidate the specific pathogenesis and treatment of GBM.

Besides methylation and IDH patterns, cell senescence is also an important factor that has been demonstrated to correlate with GBM development and the response to cancer therapy (Jinno-Oue et al., 2010; Schosserer et al., 2017; Calcinotto et al., 2019; Broestl et al., 2022). Cell senescence refers to a stable state of cell cycle arrest, in which proliferating cells are resistant to growth-promoting stimuli, usually caused by DNA damage (Childs et al., 2017). Senescent cells are characterized by morphological and metabolic changes, chromatin remodeling, altered gene expression, and the appearance of a pro-inflammatory phenotype known as the senescence-associated secretory phenotype (SASP) (Faget et al., 2019).

Although aging may have evolved as a mechanism to avoid malignant transformation of damaged cells, the onset of aging may lead to many age-related lesions, including cancer, tissue degeneration, and inflammatory diseases. Senescent cells accumulate with age, eventually leading to normal aging processes and age-related conditions (Campisi, 2013). The advances in the field of aging research have been largely driven by the connection between aging, and age-related lesions, including cancer, neurodegeneration, and metabolic and cardiovascular diseases (He and Sharpless, 2017). Rodent models have shown that selective removal of senescent cells in the body reduces inflammation and enhances immune system function, thereby delaying the progression of age-related diseases, enhancing health, and prolonging life (Gieryng et al., 2017). For example, aging-inducing drugs such as certain chemotherapy drugs may be effective against cancer by inhibiting replication potential. However, the accumulation of senescent cells in patients undergoing chemotherapy is thought to lead to adverse side effects, especially fatigue (Demaria et al., 2017). In addition, senescent cells can promote cancer recurrence and metastasis by releasing the SASP component (Guccini et al., 2021).

The results achieved with respect to the potential of existing novel GBM biomarkers are encouraging. Some of these, such as the IDH mutation, the 1p19q deletion and the methylation of the methylation of O6-methylguanine-DNA methyltransferase (MGMT) promoter, are often tested in routine clinical practice. However, there is still a lack of exploration on the biomarkers of cell senescence in GBM. Based on the importance of cell senescence for GBM, we conducted the research in this paper.

## Materials and methods

### Data collection and processing

We downloaded the expression data of The Cancer Genome Atlas (TCGA)-GBM using the UCSC xena browser (https://xena.browser.net/). The ENSG was matched to GeneSymbol, and Genes were removed when it is missing up to 50 percent of the sample. After screening, TCGA-GBM dataset contained a total of 524 samples. In addition, we obtained clinical data and sample mutation data of the corresponding samples from TCGA database through the TCGA GDC API tool. In addition, the “mRNAseq_693(batch1)” and “mRNAseq_325(batch2)” datasets containing GBM samples were downloaded from the Chinese Glioma Genome Atlas (CGGA) (http://www.cgga.org.cn/) database and the samples were screened with the same thresholds as TCGA. There are 133 and 85 glioblastoma samples left, respectively.

### Related pathways and recognition molecular subtypes of cell senescence characteristics

Here, we obtained 16 cell senescence-related pathways from the MsigDB database, and calculated the enrichment scores of each pathway using ssGSEA algorithm based on the expression data of the TCGA-GBM. In addition, we also relied on the survival package to construct a univariate COX regression model based on the clinical data of the corresponding samples to explore the relationship between pathways and prognosis (Liu et al., 2018). The consistency matrix was constructed using consistent clustering (ConsensusClusterPlus) to cluster and type the samples (Wilkerson and Hayes, 2010). The molecular subtypes of the TCGA-GBM cohort were obtained using the screened normalized enrichment score with cell senescence characteristics. We used the “pam” algorithm and the “euclidean” as metric distances and performed 500 bootstrapss procedures, each of which included 80% of the patients in the TCGA-GBM cohort. The number of clusters was set to be 2 to 10, and the optimal classification was determined by calculating the consistency matrix and cumulative distribution function (CDF), thereby determining the molecular subtypes of the TCGA-GBM cohort.

### Differences between different molecular subtypes and clinical pathological characteristics, mutation characteristics and immune characteristics

We compared the differences between clinical variable data and mutation data in TCGA-GBM cohort by molecular typing. At the same time, in order to explore the difference in immune infiltration in different molecular subtypes, we first calculated the relative abundance of 22 immune cells with the CIBERSORT algorithm, and then used the ESTIMATE algorithm to evaluate the immune cell infiltration (Aran et al., 2015; Newman et al., 2015). At the same time, we used Tumor Immune Dysfunction and Exclusion (TIDE) to assess the potential clinical effects of immunotherapy in our defined molecular subtypes, with a higher TIDE predictive score indicating a higher likelihood of immune escape, suggesting that patients were less likely to benefit from immunotherapy (Jiang et al., 2018).

### Analysis of differential pathways in different molecular subtypes

In order to investigate pathways of different biological processes in different molecular subtypes, we performed gene set enrichment analysis (GSEA) using all candidate gene sets in the Hallmark database (Liberzon et al., 2015), where we defined that false discovery rate (FDR) < 0.05 was considered to be a significant enrichment pathway. As used herein, all genes involved in hallmark pathway (h.all.v7.0.symbols.gmt) analysis were derived from MSigDB. Thus, differential pathways significantly enriched in different molecular subtypes can be explored.

### Construction of SRS.score system for evaluation of glioblastoma samples

Previous studies had demonstrated that the feasibility of gene-related prognostic models in tumors (Yuan et al., 2021a; Ren et al., 2021; Wang et al., 2022a; Yuan et al., 2022a). Differentially expressed gene in C1vs nonC1, C2vs nonC2, C3vs nonC3, C4vs nonC4 using limma package (Ritchie et al., 2015), and the threshold was set as FDR < 0.05 and |log2 fold change (FC)| >1.5. Then, the differential genes that met the threshold were subjected to univariate COX regression, and the differential genes that met the *p* < 0.01 were identified as genes with both differences and effects on prognosis. Subsequently, we used the least absolute shrinkage and selection operator (Lasso) (Yuan et al., 2021b) regression method to further screen for prognostic genes significantly related to prognosis. On the basis of linear regression, the penalty term (lambda× absolute value of slope) was added to reduce the over-fitting of the model and improve the generalization ability of the model. Here, we performed the Lasso regression using the R software package glmnet (Friedman et al., 2010). First, the change trajectory of each independent variable is analyzed, and it can be seen that with the gradual increase of lambda, the number of independent variable coefficients tending to 0 will gradually increase. We use 10-fold cross-validation to build the model and analyze the confidence interval under each lambda. The stepAIC method in Mass package starts with the most complex model and sequentially deletes a variable to reduce AIC (Zhang, 2016). The smaller the value, the better the model is. We calculated the SRS.score for each patient using the following formula: SRS.score = Σ(βi × Expi). Expi refers to the level of gene expression of phenotypic prognostic-related gene features of cell senescence, βi is the Cox regression coefficient of the correspond gene. SRS.score was normalized to zscore. According to the threshold of “0”, the patients were divided into different groups of SRS.score. The survival curve was drawn by Kaplan-Meier method for prognosis analysis, and the significance of the difference was determined by log-rank test. The genes obtained after Lasso regression were subjected to stepwise multiple factor regression analysis again, and finally determined to be the cell senescence related genes affecting the prognosis.

### Establishing and verifying the clinical prognosis model

According to the formula defined by our SRS.score system, the cell senescence-related prognosis risk score of each sample was calculated and normalized. The prognosis of each group was discussed by drawing the risk factor chart, and the time-dependent ROC curve (Blanche et al., 2013) was drawn to show the classification efficiency of 1, 2, 3 and 5-year prognosis prediction. At the same time, we use the same model to verify in CGGA cohort.

### Different clinicopathological features/immune/pathway features in SRS.score grouping

In order to explore the differences of SRS.score in the clinical data of TCGA, we grouped SRS.score according to different clinicopathological features. More, we also used CIBERSORT algorithm to calculate the relative abundance of 22 kinds of immune cells in different SRS.score groups, and ESTIMATE algorithm to evaluate the infiltration of immune cells. In order to observe the relationship between SRS.score and biological functions of different samples, we selected the gene expression profiles corresponding to glioblastoma samples in TCGA-GBM cohort and used the R software package Gene Set Variation Analysis (GSVA) (Hänzelmann et al., 2013) for ssGSEA analysis. By calculating the scores of different functions of each sample, the ssGSEA score of each sample corresponding to each function was obtained. Further, the correlation between these functions and SRS.score was calculated, and the biological functions with correlation greater than 0.3 were displayed.

### Difference in immunotherapy/chemotherapy between SRS.score groups

To explore the difference in immunotherapy between the SRS.score groups, we first compared the expression of immune checkpoints between the SRS.score groups. Again, we used TIDE to assess the potential clinical effects of immunotherapy in our defined SRS.score groups (Jiang et al., 2018).

### The combination of SRS.score and clinical pathological features further improves the prognosis model and survival prediction

We constructed the decision tree by age, sex, IDH. Mutation, MGMT. promoter.methylation, and SRS.score of glioblastoma patients in the TCGA-GBM cohort. Univariate and multivariate Cox regression analyses of SRS.score and clinical pathology were performed while nomograms were constructed to quantify risk assessment and survival probability in patients with glioblastoma. We evaluated the model’s prediction accuracy using not only Calibration curve but also decision curve analysis (DCA).

### Drug sensitivity analysis

pRRophetic (Geeleher et al., 2014) was used to predict the sensitivity of traditional medicines to IC50.

Sangerbox provided assistance with this article (Shen et al., 2022).

## Results

### Molecular typing based on cell senescence characteristics

**FIGURE 1:**
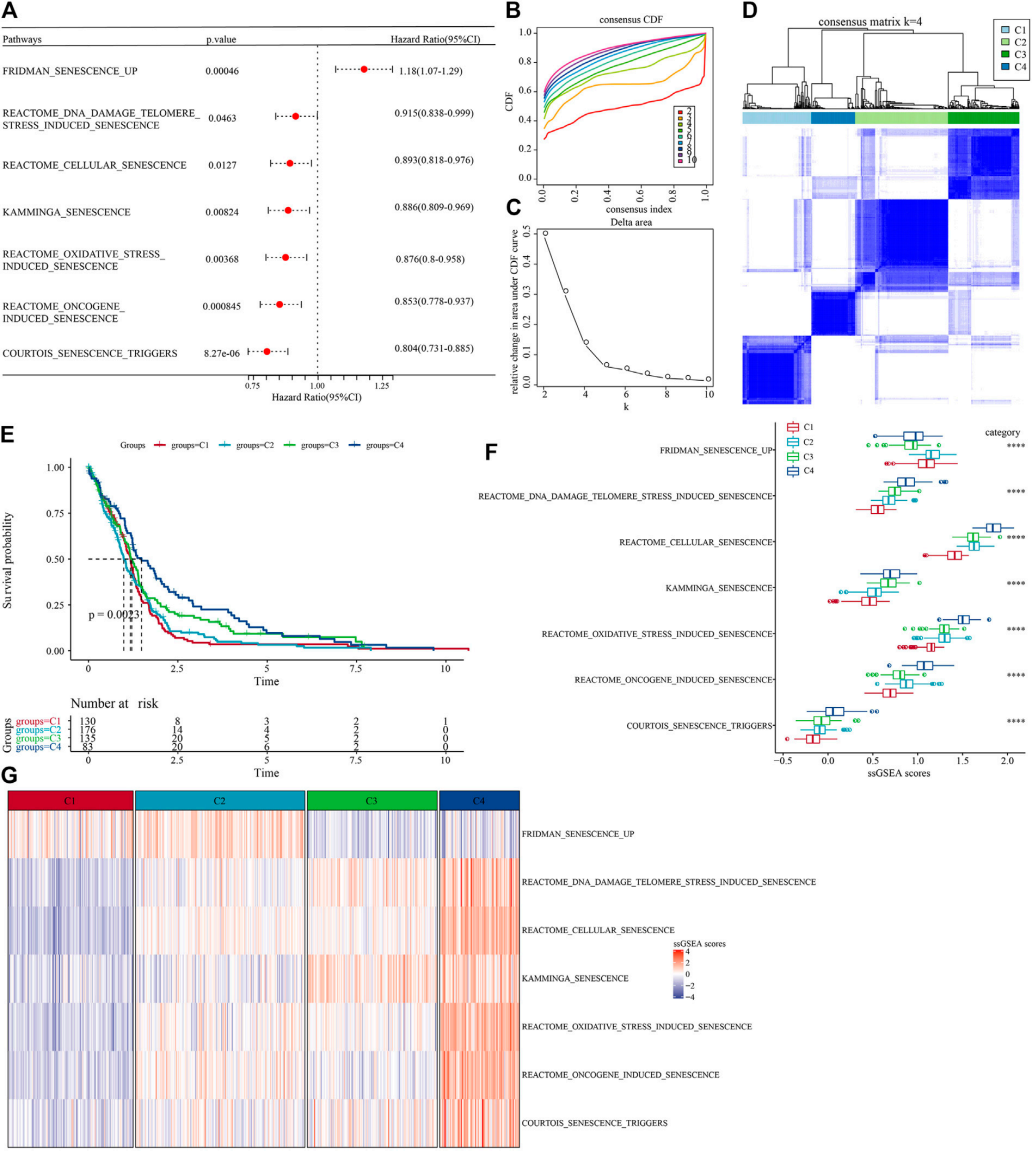
Molecular subtype with cell senescence-related pathways score in TCGA-GBM cohort. **(A)** Forest map of cell senescence associated pathways with significant prognosis; **(B)** TCGA-GBM cohort queue sample CDF curve; **(C)**. TCGA-GBM cohort sample CDF Delta area curve, Delta area curve of consensus clustering, indicating the relative change in area under the CDF curve for each category number k compared with k – 1. The horizontal axis represents the category number k and the vertical axis represents the relative change in area under CDF curve; **(D)** The heat map of sample clustering when consensus k = 4; **(E)** relationship KM curve of prognosis of four subtypes; **(F)** Differences of “ssgsea scores” of cell aging-related pathways with significant prognosis among different subtypes of TCGA-GBM cohort; **(G)** “ssGSEA scores” thermograms of cell aging-related pathways with significant prognosis in different subtypes of TCGA-GBM cohort.

The workflow was showed in Supplementary Figure S1. First, we obtained 16 cellular senescence-related pathways from the MsigDB database and calculated the enrichment scores of the 16 pathways by the ssGSEA method. Furthermore, in order to understand the relationship between these cellular senescence pathways and prognosis, we performed univariate Cox regression analysis based on clinical data from TCGA-GBM cohort. The results showed that in the TCGA-GBM cohort, a total of seven pathways were associated with the prognosis of glioblastoma, as shown in Figure 1A (*p* < 0.05). Next, we used consensus clustering analysis based on the enrichment score of seven cell senescence pathways with significant prognosis to classify samples in TCGA-GBM cohort. The optimal clustering number could be determined according to the cumulative distribution function (CDF) and observed the CDF delta area curve. From it, we could find that there was a relatively stable clustering result when the cluster was selected as 4 (Figures 1B,C). Finally, we selected k = 4 to obtain four stable molecular subtypes (Figure 1D). Further analysis of the prognostic features of these four molecular subtypes based on patient survival data revealed significant differences in their outcomes as shown in Figure 1E, where patients in the C4 had a better prognosis, while patients in the C1 subgroup had the worst prognosis (Figure 1E, *p* = 0.0023). In addition, we also compared the “ssGSEA scores” (Figures 1F,G) of the seven cell senescence-related pathways among the different molecular subtypes defined by us, and found that, except for the significant enrichment of the “fridman senescence up” pathway in C1, the enrichment scores of other cell senescence-related pathways in C4 were the highest, suggesting that the biological mechanisms related to cell senescence were significantly enriched in C4.

### Clinicopathological features between four molecular subtypes

**FIGURE 2:**
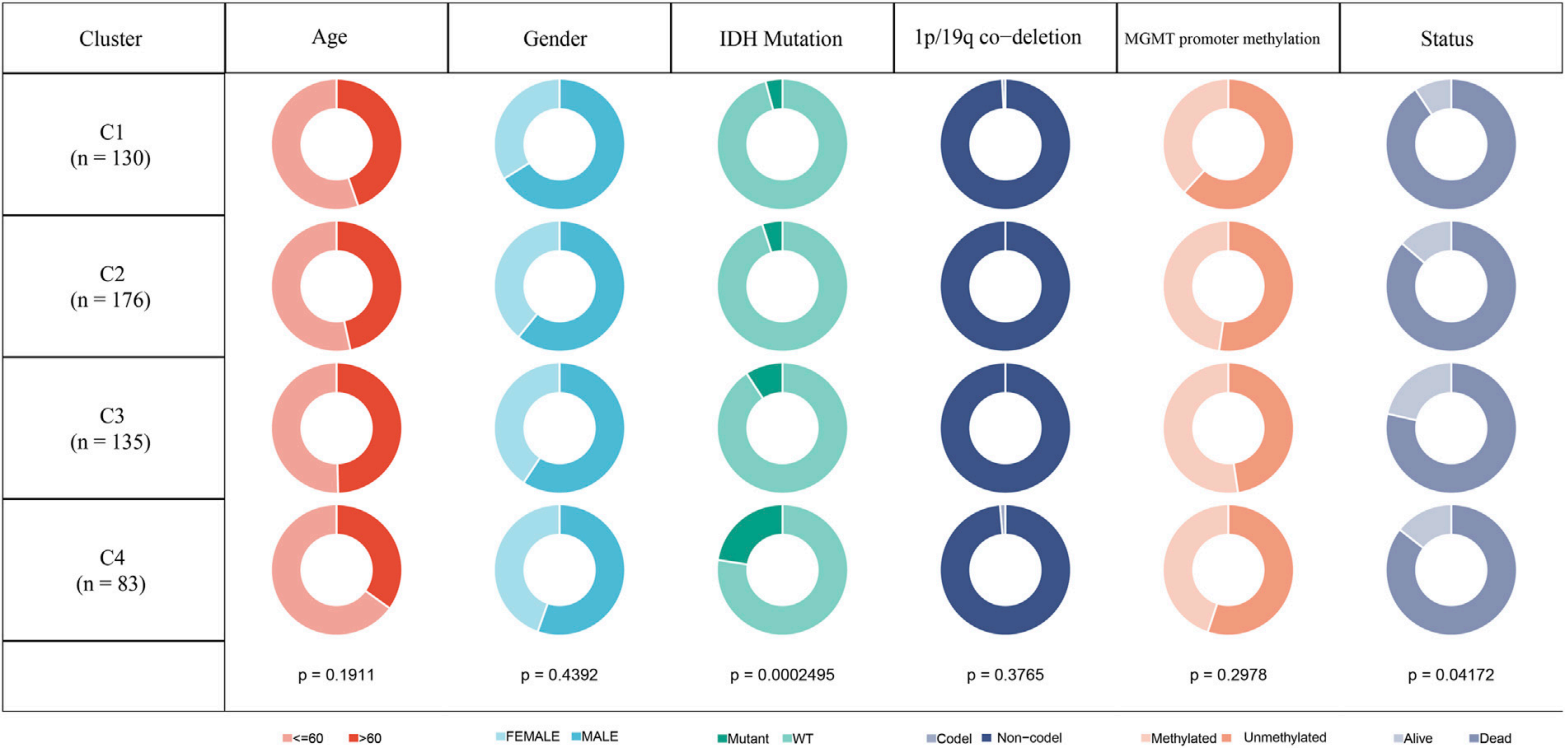
Clinicopathological features of molecular subtypes in TCGA-GBM cohort.

In the TCGA-GBM cohort, we compared the distribution of different clinical features across the four molecular subtypes to see if clinical features differed among the subtypes. The results showed that only IDH mutations and Status had significant differences in the four subtypes. The proportion of patients with IDH mutations was the highest in C4 subtype and the lowest in C1 subtype. While gender and age did not differ significantly among the four subtypes. In the aspect of 1p19q combined deletion, it can be seen that the majority of patients with glioblastoma were non-codel. As for methylation of MGMT promoter, the methylation degree of MGMT promoter of subtype C1 was significantly lower than that of other molecular subtypes (Figure 2).

### Mutation characteristics between molecular subtypes

**FIGURE 3:**
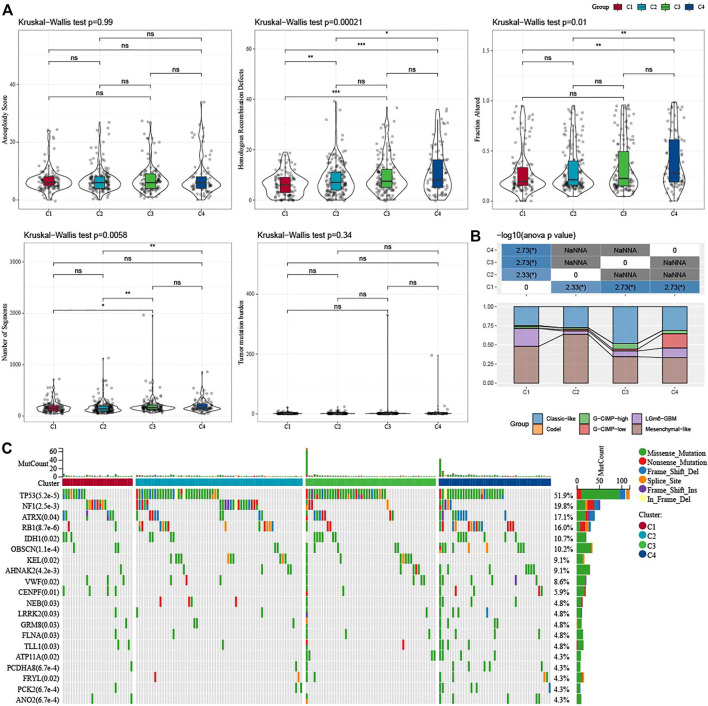
Genomic changes of molecular subtypes in TCGA-GBM cohorts. **(A)** Comparison of the differences among the molecular subtypes of homologous recombination defects, aneuploidy score, fraction altered, number of segments, and tumor mutation burden in the TCGA-GBM cohort; **(B)** Comparison of four molecular subtypes with other molecular subtypes; **(C)** Somatic mutations in four molecular subtypes (chi-square test). *p < 0.05; **p < 0.01; ***p < 0.0001; ****p < 0.0001.

In addition, we explored differences in genomic changes between the four molecular subtypes in the TCGA cohort. Here, we obtained the molecular characteristics of TCGA-GBM from a previous pan-cancer study (Thorsson et al., 2018) and performed multi-group analysis of different molecular subtypes using the Kruskal–Wallis test. Lower scores of Homologous Recombination Defects and Fraction Altered were shown in C1 (Figure 3A). We also compared the relationships of the 4 molecular subtypes with the 6 molecular subtypes reported in the previous literature (Figure 3B), and found that there were more “Classic-like” molecular subtype in C3, more “Mesenchymal-like” molecular subtype in C2, and more “LGm6-GBM” in C1. In addition, we also analyzed the correlation between gene mutation and molecular subtypes (Figure 3C), and found that there was a significant correlation between molecular subtypes and gene mutation. The TP53, NF1, ATRX, RB1, and IDH1 genes have a wide range of somatic mutations in glioblastoma, with the IDH1 gene having a high mutation frequency in both C3 and C4. The mutation rate of NF1 in C1/C2 is relatively high, while that in C3/C4 is relatively low. The association between NF1 and GBM has been well studied and it is interesting to note that some changes, such as inactivated NF1 mutations/deletions, are associated with estimated proportion of immune cells or cluster activities (Luoto et al., 2018).

### The immune characteristics between molecular subtypes

**FIGURE 4:**
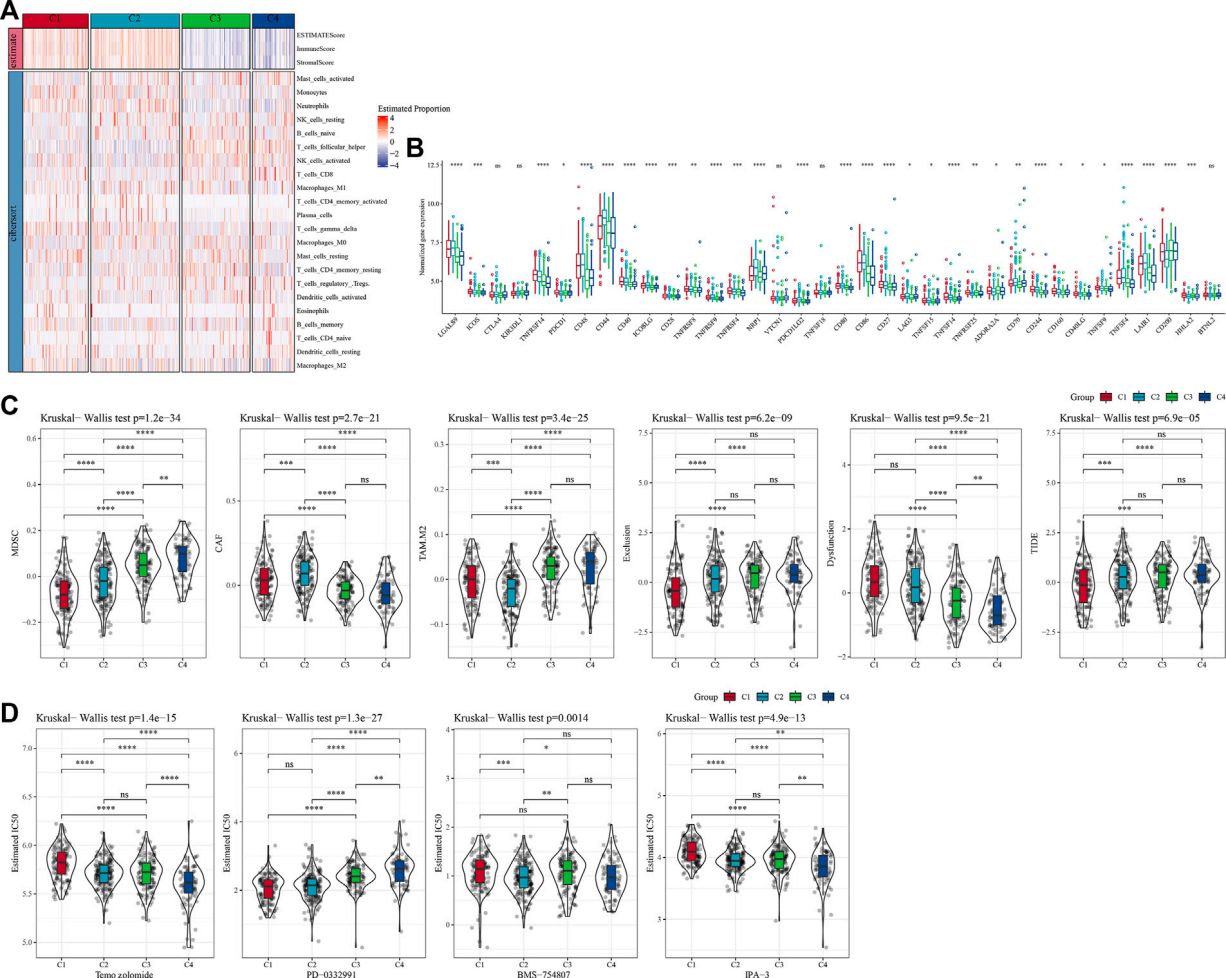
Differences of immune cell infiltration in different molecular subtypes. **(A)** The differences of 22 immune cell scores among different molecular subtypes in the TCGA-GBM cohort; The differences of ESTIMATE immune infiltration among different molecular subtypes in the TCGA-GBM cohort; **(B)** Immunocheckpoints for differential expression between different subgroups in the TCGA-GBM cohort; **(C)** Difference in TIDE analysis results between groups in the TCGA-GBM cohort; **(D)** The box plots of the estimated IC50 for temozolomide, PD-0332991, BMS-754807 and IPA-3 in TCGA-GBM cohort.

To further clarify the differences in the immune microenvironment of patients in different molecular subtypes, we assessed the degree of immune cell infiltration in our TCGA-GBM cohort by the expression levels of immune-related genes. First, we calculated the relative abundance of 22 immune cells using CIBERSORT. For example, in Figure 4A, we could observe that most immune cell types showed significant differences between molecular subtypes. The most significantly different immune cells included activated CD4 memory T cells, follicular helper T cells, gamma delta T cells, and neutrophils. At the same time, we also used ESTIMATE to assess immune cell infiltration. “ImmuneScore” of C1 and C2 is significantly higher than that of other subtypes, indicating high immune cell infiltration. More than that, we analyzed whether there were differences in immunotherapy between different molecular subtypes in the TCGA-GBM cohort. First, we compared the expression of immune checkpoints among subtypes (Figure 4B), and we could see that most of the immune checkpoints were differentially expressed among four subtypes. Further, we analyzed the differences in immunotherapy among the different subtypes. Here, TIDE was conducted to assess the potential clinical effects of immunotherapy in our defined molecular subtypes. A higher TIDE predictive score indicates a higher likelihood of immune escape, suggesting that patients are less likely to benefit from immunotherapy. As shown in Figure 4C, TIDE score was the lowest in C1 of the TCGA-GBM cohort and it was more likely to benefit from immunotherapy. At the same time, we also observed a high dysfunction score for subtypes C1 and C2, which indicates that although C1 and C2 had high immune infiltration, it was the cause of poor prognosis due to dysfunction. In addition, we also analyzed the response levels of different molecular subtypes in the TCGA cohort to the common chemotherapeutic or targeted drugs Temozolomide, PD-0332991, BMS-754807 and IPA-3, and found that C1 was more sensitive to PD-0332991, C2 subtype was more sensitive to BMS-754807, and C4 was more sensitive to Temozolomide and IPA-3 (Figure 4D).

### Pathway analysis in four molecular subtypes

**FIGURE 5:**
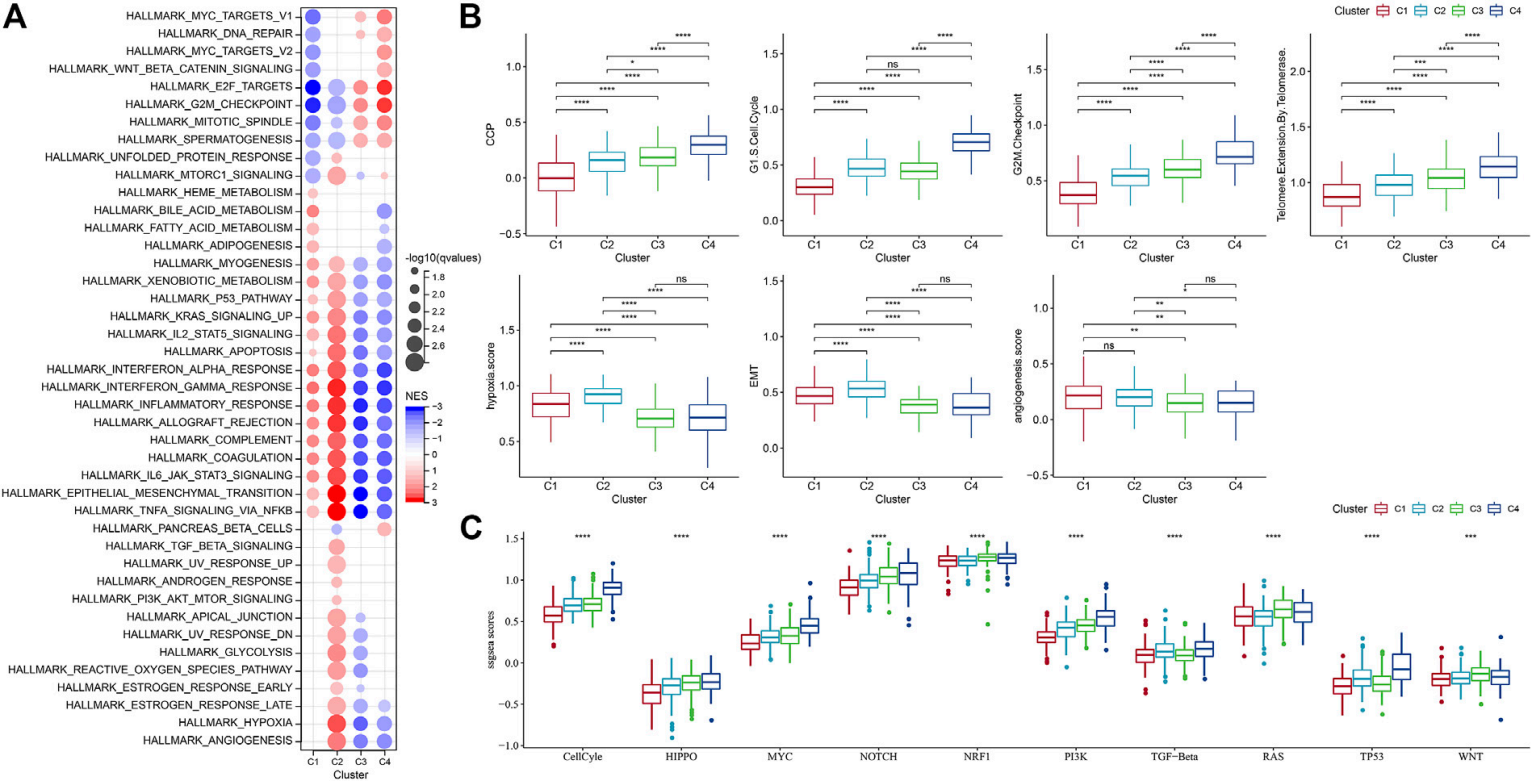
Differences in pathway enrichment fractions among different molecular subtypes. **(A)** GSEA analysis results from the TCGA-GBM cohort; **(B)** Differences in ssGSEA scores of cell senescence-associated pathways in the TCGA-GBM cohort; **(C)** Score differences of different molecular subtypes in 10 pathways related to tumor abnormalities in the TCGA-GBM cohort.

Next, we analyzed whether there were differentially activated pathways among different molecular subtypes. To identify these pathways, we performed GSEA using all candidate gene sets from the Hallmark database (Liberzon et al., 2015), where FDR < 0.05 was considered to be significantly enriched. We obtained a total of 42 pathways, and by observing the normalized enrichment score (NES), we could see that the enrichment scores of most of the pathways in C3 and C4 in the TCGA-GBM cohort were less than 0, which represented that most of the pathways were likely in an inhibitory state compared with C1 and C2. Compared to the C3, 27 pathways were significantly enriched in the C1 and 35 pathways were significantly enriched in the TCGA-GBM cohort as shown in Figure 5A. We also compared abnormal pathways between C1 and C3 in different cohorts of glioblastomas, as shown in Figure 5B. On the whole, the activated pathways mainly include some immune-related pathways such as interface gamma response, interface alpha response, allograft rejection, and inflammatory response. Other cell cycle-related pathways were also activated, such as E2F targets, G2M checkpoint, and MYC targets v1 (Figure 5A). In addition, we also compared the pathways of difference between C1 and C2, between C1 and C3, and between C2 and C3 among different TCGA-GBM cohorts (Figure 5C). All in all, the immune regulatory pathway and cell cycle pathway of patient C1 were activated, so we deduced that the cell senescence gene for molecular typing might play a very important role in the immunosuppressive microenvironment and tumor microenvironment. Next, we analyzed whether there were differentially activated pathways among different molecular subtypes. To identify these pathways, we performed GSEA using all candidate gene sets from the Hallmark database, where FDR < 0.05 was considered to be significant enrichment. A significant accumulation of 29 pathways in subtype C1 was seen in the TCGA-GBM cohort, and overall, the activated pathways mainly included some immune-related pathways such as interferon alpha response, interferon gamma response, inflammatory response, allograft reject, and complement pathway. (Figure 5A). We also compared the pathways of differences between different C2 subtypes and other subtypes, and we could find that the immune regulatory pathway of patients with C2 subtype was in the active state. In addition, we found that C3 and C4 subtypes were generally immunosuppressed, while cell cycle-related pathways were activated.

It has been found in a previous study (Wang et al., 2022b) that cancer cells can induce cell senescence by inhibiting cell cycle, and a major feature of senescent cells is the up-regulation of cyclin-dependent kinases (CDK inhibitory proteins), such as InK4a and p21, to induce cell cycle arrest. Thirty-one genes related to cell cycle progression (CCP) were identified in a previous study (Cuzick et al., 2011). CCP scores of samples in the TCGA-GBM cohort were calculated by ssGSEA method, and the results showed that CCP scores of C4 were significantly higher than those of other molecular subtypes. At the same time, we calculated the G1/S score of each sample in the TCGA-GBM cohort by downloading G1/S phase-related genes from KEGG official website. Similarly, we used ssGSEA’s method to calculate the score of each sample in the TCGA data set about G2 checkpoint. It turned out that both G1/S and G2 checkpoints had significantly higher C4-related scores than the other subtypes (Figure 5B).

Similarly, inhibition of telomerase will also induce cell senescence (Wang et al., 2022b). In the body, cancer cells usually avoid telomere loss by activating telomerase activity. Reactome telomere extension by telomerase was download *via* GSEA. The reaction cycle has been inferred from *in vitro* studies of telomerase from multiple organisms (Vega et al., 2003; Smogorzewska and de Lange, 2004). Here, the score was calculated using the ssGSEA method, and we finally found that the telomere extension score of telomerase in C4 was higher than that of other subtypes. Aging cells secrete cytokines that affect the surrounding cells. This effect can be achieved by promoting epithelial-mesenchymal transition (EMT) to facilitate tumor migration and metastasis. Moreover, aging tumor cells can recruit special macrophages to promote the production of blood vessels and lymphatic vessels, and provide other tumor cells with the oxygen and nutrients needed for growth, thereby promoting the growth and metastasis of tumors. It was found that the EMT score of C1 was higher than that of other subtypes (Yu et al., 2021). We selected genes in the hypoxia pathway to analyze the hypoxia score of the samples with ssGSEA. Meanwhile, we analyzed the angiogenesis scores of the samples based on 24 genes selected from the literature (Masiero et al., 2013), and found that the hypoxia and angiogenesis scores of C1 and C2 were significantly higher than those of the other two molecular subtypes. In addition, we also analyzed the differences of four molecular subtypes in the previous study (Sanchez-Vega et al., 2018) of the 10 oncogenic pathways, and significant differences in the 10 pathways could be observed. The ssGSEA scores of some famous pathways, such as Cell Cycle, HIPPO, MYC, also were calculated and we found different subtypes had evident distinctions among them.

### Determination of key genes related to cell senescence characteristics

**FIGURE 6:**
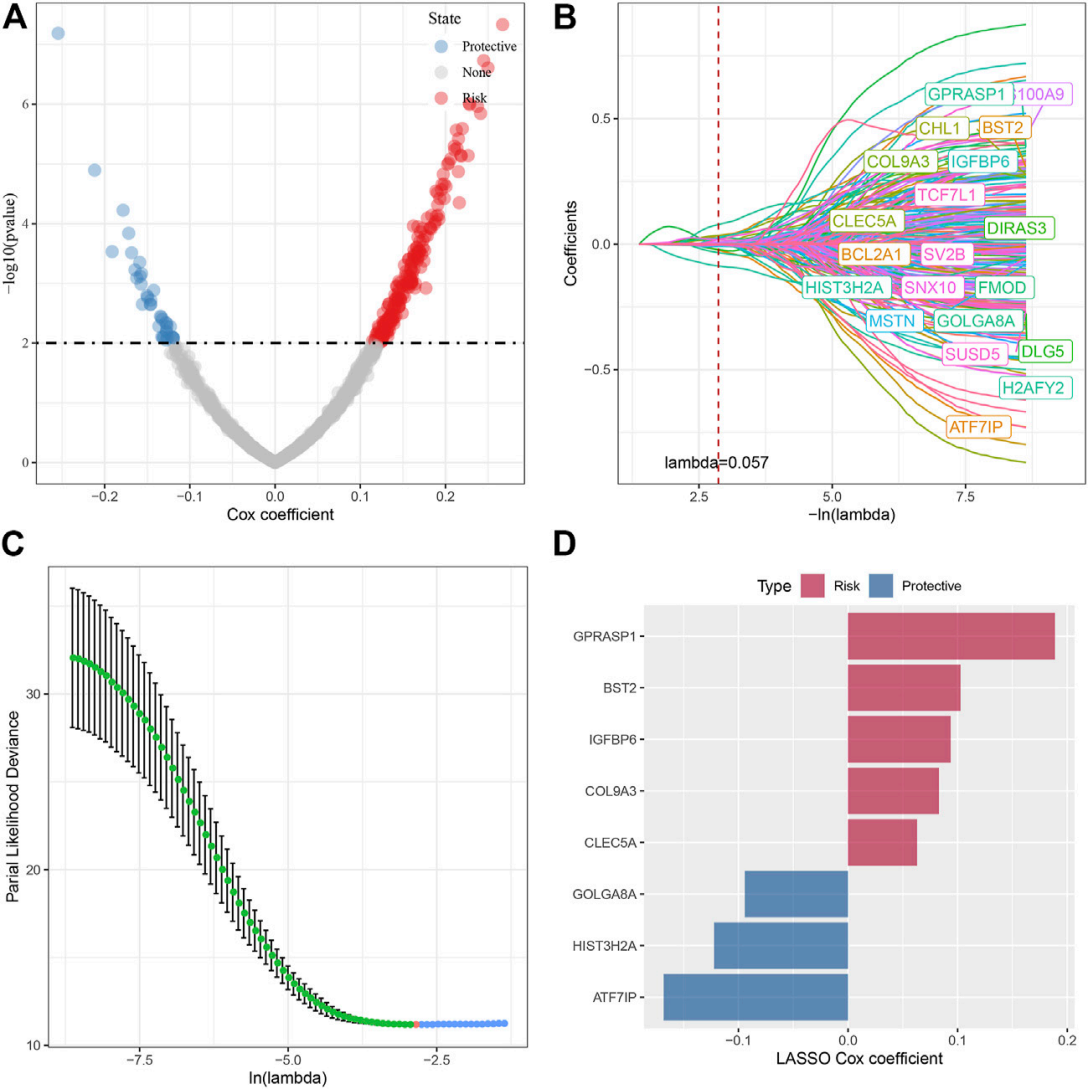
Screening of key genes in cell senescence-related pathways. **(A)** A total of 232 promising candidates were identified among the DEGs; **(B)** The path of each argument as lambda changes; **(C)** Confidence interval under lambda. **(D)** Distribution of lasso coefficients of the senescence-related gene signatures.

In the previous analysis, we identified four different molecular subtypes based on the enrichment score of seven cell senescence-related pathways significantly associated with prognosis. Next, we screened the genes with differential expression between C1 and non-C1, C2 and non-C2, C3 and non-C3, and C4 and non-C4 subtypes (FDR < 0.05 and |log2FC| > 1.5). There was 197 DEGs in C1 vs nonC1, 313 DEGs in C2 vs nonC2, 331 DEGs in C3 vs nonC3, 718 DEGs in C4 vs nonC4. Through union of 4 molecular types, 1005 genes were finally screened. Next, we performed univariate COX regression analysis on the differentially expressed genes among subtypes, and identified a total of 232 genes that had a greater impact on the prognosis (*p* < 0.01), including 194 Risk and 38 Protective genes (Figure 6A). Further, we used lasso regression to further compress these 232 cell senescence genes to reduce the number of genes in the risk model. From the Figure 6, we can see that the model is optimal when lambda = 0.057. Therefore, we chose 20 genes when lambda = 0.057 as the target gene for the next step (Figures 6B,C). Further, based on the 20 genes in the lasso analysis results, we use the stepwise multivariate regression analysis, and the stepwise regression utilizes the AIC chi-square information criterion, which considers the statistical fitting degree of the model and the number of parameters used for fitting. It indicates that the model obtains enough fitting degree with fewer parameters. Finally, we identified eight genes (GPRASP1, BST2, IGFBP6, COL9A3, CLEC5A, GOLGA8A, HIST3H2A, ATF7IP) as cell senescence-associated genes that affect prognosis (Figure 6D). RiskScore = +0.094*IGFBP6+0.063*CLEC5A+0.189*GPRASP1-0.094*GOLGA8A+0.083*COL9A3-0.168*ATF7IP+0.103*BST2-0.122*HIST3H2A.

### The establishment and verification of clinical prognosis model

**FIGURE 7:**
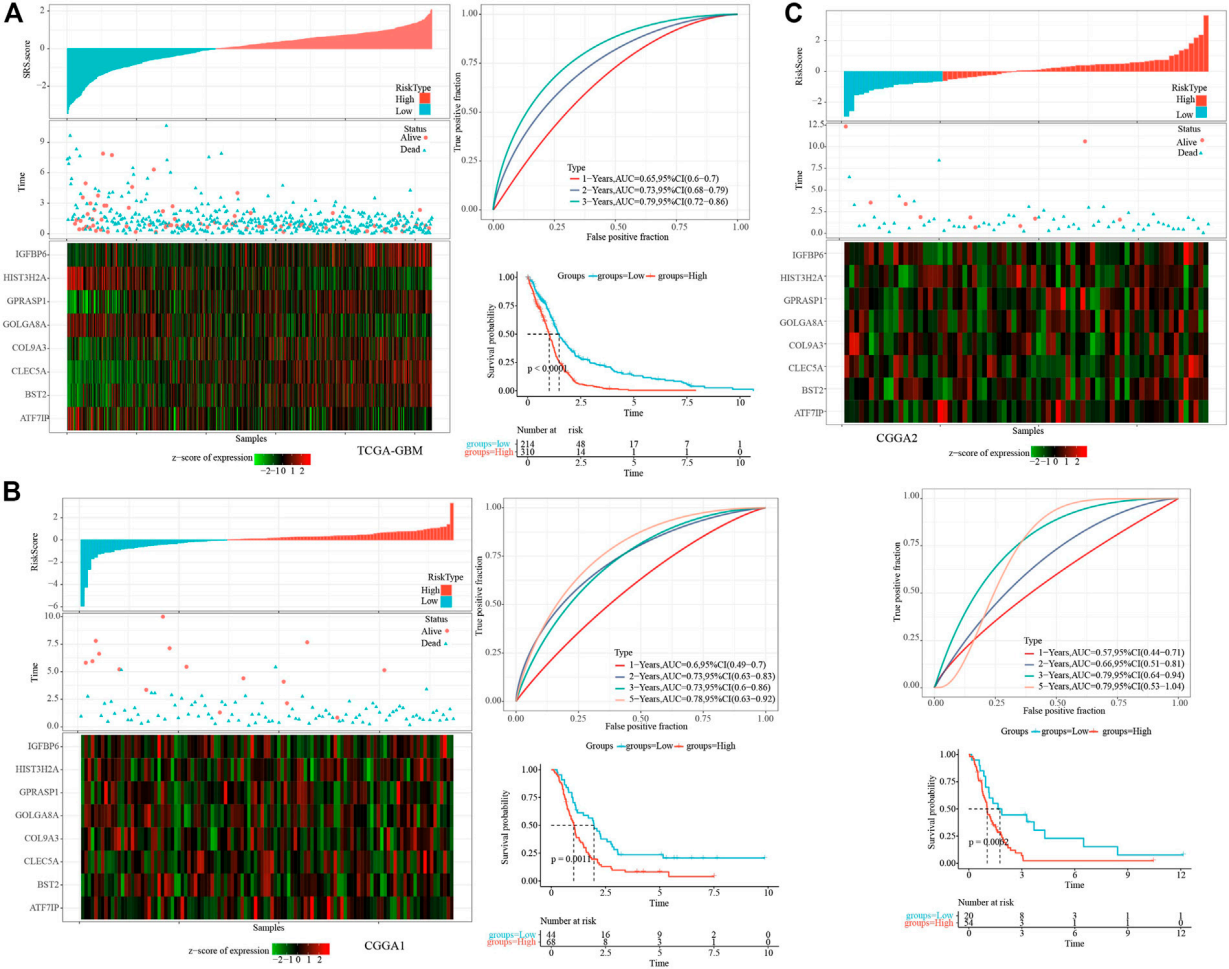
Establishment and test of SRSscore model. **(A)** SRS.score, survival time and survival state, and expression of senescence-related genes in the TCGA-GBM cohort; ROC curve and AUC of the SRS.score classification in the TCGA-GBM cohort; KM survival curve distribution of SRS.score in the TCGA-GBM cohort; **(B)** SRS.score, survival time and survival state, and expression of senescence-related genes in the CGGA1 cohort; ROC curve and AUC of the SRS.score classification in the CGGA1 cohort; KM survival curve distribution of SRS.score in the CGGA1 cohort; **(C)** SRS.score, survival time and survival state, and expression of senescence-related genes in the CGGA2 cohort; ROC curve and AUC of the SRS.score classification in the CGGA2 cohort; KM survival curve distribution of SRS.score in the CGGA2 cohort.

The cell senescence-related prognostic risk score (SRS.score) for each sample was calculated and normalized according to the formula defined by the SRS.score of our samples. The distribution of SRS.score for patients in the TCGA-GBM cohort was displayed by mapping risk factors, suggesting that a higher SRS.score sample had a worse prognosis. 1-, 2- and 3- years AUC were 0.65, 0.73, and 0.79, respectively, and low group had better survival time in TCGA dataset (Figure 7A). To confirm the robustness of the clinical prognosis model prediction of cell senescence-associated gene signature, we performed verification in two CGGA cohorts, and we calculated the SRS.score of patients in two CGGA cohorts in the same way. The distribution of SRS.score, status and gene expressions were showed in CGGA1 dataset. 1-, 2- , 3- and 5- years AUC were 0.6, 0.73, 0.73, and 0.78, and low group had better survival time in CGGA1 dataset (Figure 7B). In the CGGA2 cohorts, similarly phenomenon were observed (Figure 7C).

### The manifestations of SRS.score in different clinical pathological features

**FIGURE 8:**
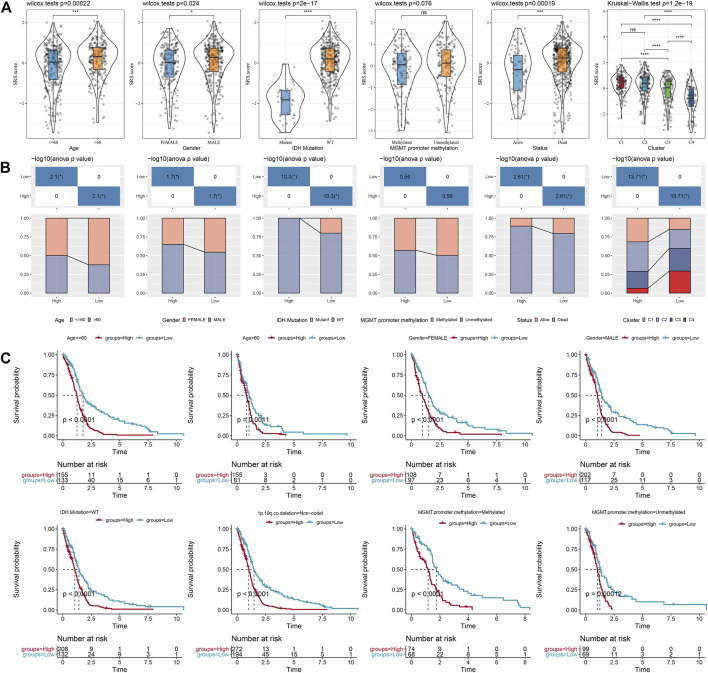
Specificity of clinical features in the SRS.score subgroups. **(A)** Differences of SRS.score among different clinical pathological groups in TCGA-GBM cohort; **(B)** Clinicopathological features between SRS.score groups in TCGA-GBM cohort; **(C)** KM curve of SRS.score between high and low risk groups among different clinical pathological groups in TCGA-GBM cohort.

We compared the distribution of SRS.score among the subgroups with clinicopathological features in the TCGA-GBM cohort and found significant differences between the SRS.score subgroups for age, gender, and IDH Mutation. At the same time, we compared the differences of SRS.score among molecular subtypes and found that SRS.score of C1 with poorest prognosis had the highest SRS.score while C4 had the lowest SRS.score with best prognosis. Meanwhile, we also compared the differences in clinical pathological characteristics between the SRS.score subgroups in the TCGA-GBM cohort, and found that patients in the high SRS.score group were older and accounted for more proportion of male patients. In addition, we also compared the relationship between the SRS.score subgroup and the four previously defined molecular subtypes, and found that patients with C1 of SRS.score-high were significantly higher than those with SRS.score-low group (Figures 8A,B). In addition, we compared the presence of different clinicopathological features in the TCGA-GBM cohort for differences in prognosis in our defined high-and low-risk groups for SRS.score, and the results showed that our risk group performed equally well in different clinical subgroups, demonstrating the reliability of our risk group (Figure 8C).

### Immune/pathway characteristics between SRS.score subgroups

**FIGURE 9:**
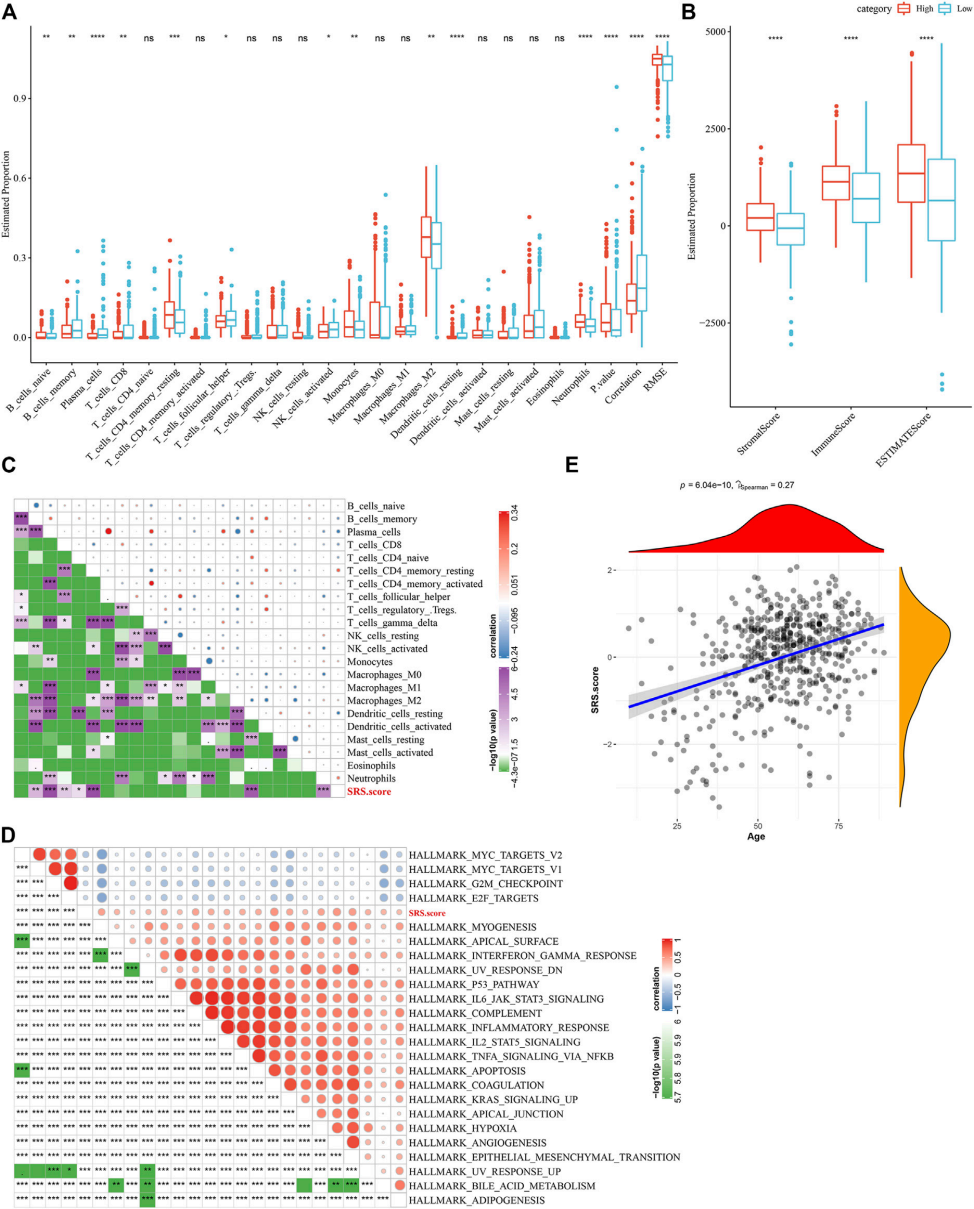
Difference between immune infiltration and Hallmarker pathway in SRS.score subgroups. **(A)** The proportion of immunocytes in the TCGA-GBM cohort; **(B)** The proportion of immunocyte components calculated by ESTIMATE software in the TCGA-GBM cohort; **(C)** Correlation analysis of 22 immune cell components and SRS.score in TCGA-GBM cohort; **(D)** Results of correlation analysis between KEGG pathway with correlation greater than 0.3 with SRS.score and SRS.score; **(E)** Analysis of Correlation between Age of Samples and SRS.score in the TCGA-GBM cohort.

To elucidate the differences in the immune microenvironment of patients in the SRS.score subgroup, we compared the relative abundance of 22 immune cells in the SRS.score high and low subgroups in the TCGA-GBM cohort and observed that most of the immune cells differed significantly in the SRS.score high and low subgroups. The immunocytes of neutrophils, plasma cells, and dendritic cells resting showed the most significant differences in the SRS.score subgroups, indicating that the difference in SRS.score might be due to the difference in the infiltration degree of these immunocytes (Figure 9A). Meanwhile, ESTIMATE was conducted to evaluate the immune cell infiltration. “ImmuneScore” in the SRS.score-high group was significantly higher than that in the SRS.score-low group, with higher immune cell infiltration (Figure 9B). Furthermore, we analyzed the relationship between SRS.score and 22 immune cell components, and we found a significant correlation between SRS.score and some immune cells (Figure 9C). In order to observe the relationship between SRS.score and biological function of different samples, we selected the gene expression profiles corresponding to glioblastoma samples in the TCGA-GBM cohort, and performed ssGSEA analysis. For the functions with correlation greater than 0.3, a total of 23 pathways significantly correlated with SRS.score were obtained, of which four pathways were negatively correlated with SRS.score, and the remaining 19 pathways were positively and negatively correlated with SRS.score (Figure 9D). In addition, we also calculated the correlation between SRS.score and the patient’s age, and found a significant positive correlation between SRS.score and the patient’s age (Figure 9E).

### Differences of immunotherapy/chemotherapy between SRS.score subgroups

**FIGURE 10:**
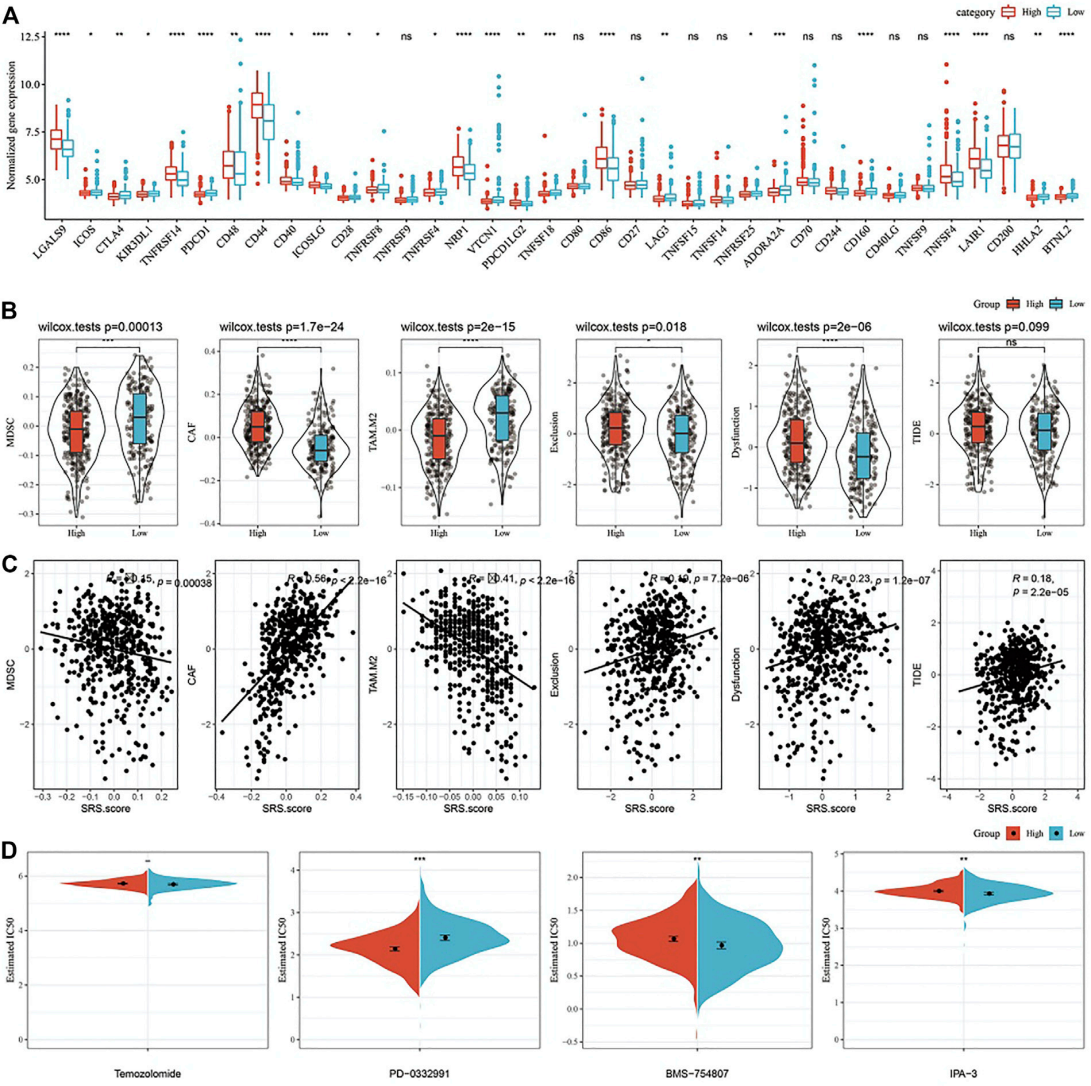
Differences in Immune Checkpoints and Drug Susceptibilities in the SRS.score subgroups. **(A)** Immune checkpoints of differential expression between different groups in TCGA-GBM cohort; **(B)** The difference of TIDE analysis results between different packets in TCGA-GBM cohort; **(C)** Correlation analysis between tide results and SRS.score; **(D)** The box plots of the estimated IC50 for cisplatin, doxorubicin, methotrexate and paclitaxel in TCGA-GBM cohort.

Furthermore, we analyzed whether there were differences in immunotherapy among SRS.score groups in TCGA-GBM cohort. On the whole, the transcription level of immune checkpoint related genes in SRS.score-high is significantly higher than that in SRS.score-low (Figure 10A). Further, we analyzed the differences of different SRS.score groups in immunotherapy. MDSC, the myeloid-derived suppressor cells, is the precursor of dendritic cells (DCs), macrophages and granulocytes, and has the ability to significantly inhibit the immune cell response. We found that the level of MDSC in SRS.score-Low was significantly higher than that in SRS.score-high (*p* = 0.00013). M2 tumor-associated macrophages have the same trend as MDSC. On the contrary, the scores of cancer associated fibroblasts (CAF), Exclusion and Dysfunction in SRS.score-high are significantly higher than those in SRS.score-low (*p* < 0.05). Here, we use TIDE to evaluate the potential clinical effects of immunotherapy in our defined SRS.score-high and low groups. The higher the TIDE prediction score, the higher the probability of immune escape, suggesting that patients are less likely to benefit from immunotherapy. Furthermore, we analyzed the relationship between SRS.score and TIDE score, and found that there was a significant positive correlation between SRS.score and TIDE score (*p* < 0.001), which suggested that SRS.score-high group had a higher possibility of immune escape and a lower possibility of benefiting from immunotherapy. There is a significant positive correlation between SRS.score and MDSC, tumor-associated macrophage (TAM).M2 and Exclusion (Figures 10B,C). In addition, we also analyzed the response of SRS.score in TCGA-GBM cohort to traditional chemotherapy/target drugs temozolomide, PD-0332991, BMS-754807, and IPA-3, and found that SRS.score-low was more sensitive to BMS-754807 and IPA-3. SRS.score-high is more sensitive to PD-0332911, which indicates that the effect of using PD-0332911 as a chemotherapy drug may be better than other chemotherapy drugs for GBM patients (Figure 10D).

### SRS.score combined with clinicopathological features to further improve the prognosis model and survival prediction

**FIGURE 11:**
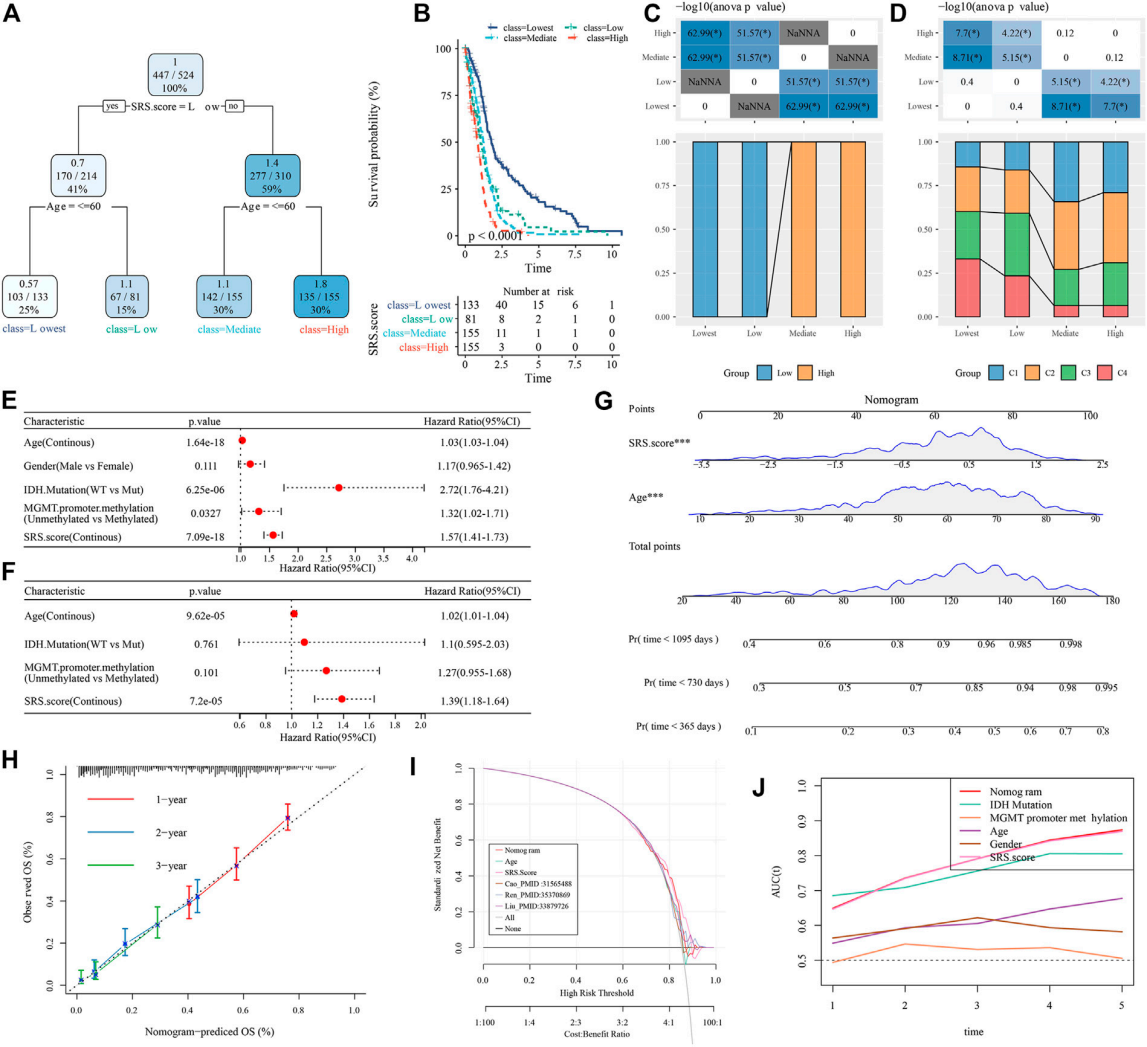
Identify the clinical features that have the greatest impact on prognosis in GBM. **(A)** Patients with full-scale annotations including SRS.Score, age, gender , IDH.Mutation and MGMT.promoter.methylation were used to build a survival decision tree to optimize risk stratification; **(B)** Significant differences of overall survival were observed among the four risk subgroups; **(C,D)** Comparative analysis in subgroups; **(E,F)** Univariate and multivariate analysis of SRS.score and other clinical features; **(G)** Nomogram model of SRS.Score and age; **(H)** 1-,2-and 3-year calibration curves for nomograms; **(I)** Decision curve of nomogram; **(J)** Nomograms showed the strongest viability predictions.

Here, we constructed a decision tree according to the age, sex, IDH.Mutation, MGMT.promoter.methylation and SRS.score of glioblastoma patients in TCGA-GBM cohort. The results showed that only two key factors, SRS.score and age, remain in the decision tree. According to these two key factors, four different risk subgroups (Figure 11A) are determined. SRS.score is the most powerful parameter. There were significant differences in overall survival in the four risk subgroups. Among them, the risk subgroups “Lowest” and “Low” were all patients with SRS.score-low. The prognosis in the class = High group was the worst, which indicated that the prognosis of older patients with high SRS.score was even worse (Figures 11B,C). In addition, we also found differences in the distribution of our defined molecular subtypes among the different risk subgroups, with the “Mediate” and “Highest” risk subgroups accounting for more of our defined molecular subtypes C1 and C2 (Figure 11D). Univariate and multivariate Cox regression analyses of SRS.score and clinical pathology revealed that SRS.score was the most significant prognostic factor (Figures 11E,F). In order to quantify the risk assessment and survival probability in patients with glioblastoma, we combined SRS.score with other clinical pathological features to establish nomogram, and from the model results, SRS.score had the greatest impact on the survival rate prediction (Figure 11G). Further, we evaluated the prediction accuracy rate of the model using Calibration curve. We could observe that the predicted calibration curves of the three calibration points in 1, 2, and 3 years were close to the coincidence with the standard curve, which indicated that the nomogram had good prediction performance (Figure 11H). In addition, DCA was used to evaluate the reliability of the model. It can be observed that the benefits of SRS.score and nomogram are significantly higher than the extreme curve. Compared with other clinical pathological features, Nomogram and SRS.score both show the most powerful survival prediction ability (Figures 11I,J).

## Discussion

Glioblastoma is the most malignant glioma of astrocytomas. At present, the standard treatment is mainly surgical resection, postoperative adjuvant chemotherapy and radiotherapy. However, tumor recurrence occurs in approximately 90% of patients 6–9 months after treatment. With the development of next-generation sequencing (NGS) technology, we have obtained a better understanding of glioblastoma. The process of cell aging is closely related to the aging and diseases of the body. With the deepening of the research on cell aging, it is found that all kinds of stimulation to cells, stress reaction and DNA damage may cause cell aging. It has been suggested that after activation of oncogenes, telomere dysfunction can be induced in certain cells with precancerous lesions, accelerating the production of cell senescence. With the deepening of research, the phenomenon of oncogene-induced cell senescence has been considered as an important barrier against tumorigenesis *in vivo*, and there is a complex correlation between cell senescence and the occurrence/development of tumors. Therefore, we sorted out 16 different pathways related to cell aging, screened out 7 pathways related to prognosis through the clinical data of TCGA, and identified 4 molecular subtypes through consistent clustering.

We explored the clinicopathological features in four molecular subtypes. We found that IDH mutation is abundant in C4 subtypes, and that the prognosis of C4 subtype is better than that of other subtypes. As we know from the previous literature, the IDH mutation is an early event in the development of glioma and it persists throughout the development from diffuse and anaplastic astrocytoma of the IDH mutation to GBM. Gliomas with IDH1 and IDH2 mutations had a better prognosis than wild-type gliomas (Cohen et al., 2013). 1p19q codeletion represents the combined loss of chromosome 1 (1p) and chromosome 19 (19q) in short arm, which is considered as a genetic marker to predict the response of patients with diffuse glioma to chemotherapy and combined radiotherapy and chemotherapy, and the overall survival time of patients with diffuse glioma is longer (Louis et al., 2019; Zheng et al., 2020). In this study, it was found that the mutation probability of NF1 gene in C1 and C2 with worse prognosis was high. According to previous reports in the literature, NF1 mutation affected the drug resistance of targeted drug mechanism of cerebellar glioblastoma (Cho et al., 2019). Six of the seven senescence pathways with prognostic significance have protective factors for patients, except one: fridman senescence up. This risk pathway was significantly enriched in C1, and the other six protective pathways were most significantly enriched in C4. This is probably the reason for the worst prognosis of C1. The process by which cancer cells bypass cellular senescence and become immortal requires not only the loss of a key gene such as TP53, but also additional mutations and/or epigenetic changes.

Homologous recombination defects and fraction altered are more common in C1. The absence of homologous recombination leads to genetic instability, genomic instability being the hallmark of various cancers, with increasing accumulation of DNA damage. The application of radiotherapy and chemotherapy in cancer treatment is generally based on this property of cancer (Lim et al., 2012; Huang and Zhou, 2021; Matos-Rodrigues et al., 2021). Fraction of genome altered is the percentage of genome that has been affected by copy number gains or losses. Moreover, the variation of copy number will also affect the prognosis of GBM patients (Umehara et al., 2019).

The immune microenvironment plays an important role in tumors, with infiltrating immune cells in GBM composed of central nervous system (microglia) and peripheral macrophages, granulocytes, bone marrow-derived suppressor cells (MDSC), and T lymphocytes. Intratumoral density of glioma-associated microglia/macrophages (GAMs) and MDSC was the highest among malignant gliomas and negatively correlated with patient survival (Gieryng et al., 2017). The immune scores of C3 and C4 with better prognosis were much lower than those with worse prognosis, which indicated that with the significant activation of cell senescence pathway, GBM could be induced to age and tumor growth could be inhibited, just as TGF-β-induced p15INK4B expression accelerated the aging of liver cancer (Senturk et al., 2010). With the MDSC score in C4 being the highest, it suggested immune infiltration was in an activated state in contrast to C1. C1 had the lowest TIDE score and was associated with the worst prognosis. It indicated that while there were immunologic opportunities in C1, it was also the most likely to respond to immunotherapy, thereby improving the prognosis. Among several chemotherapeutic agents, PD-0332991 was more effective as a chemotherapy strategy for patients in C1.

Among the differential pathways of Hallmark, most of the pathways in C1 and C2 were significantly up-regulated (NES > 0), such as IL2-JAK-STAT3 signaling, TNFA signaling *via* NFKB, and complement, while these pathways were significantly down-regulated in C3 and C4 (NES < 0). STAT3 expression in tumor-associated immune cells was also exceptionally strong. Continuous activation of STAT3 in tumor-associated immune cells activates the expression of downstream genes VEGF, IL-10 and IL-6, and causes the proliferation of tumor-infiltrating hematopoietic stem cells, which ultimately leads to poor prognosis (Wang et al., 2018). Interestingly, CCP scores, G1/S phase and G2 checkpoint-related scores of C4 were much higher than those of other subtypes, indicating that cell cycle-related pathways were significantly activated in C4.

Previous researches reported the significance of gene signature in cancer (Yuan et al., 2021c; Yuan et al., 2022b; Chen et al., 2022; Ma et al., 2022; Miao et al., 2022; Ren et al., 2022). In this study, we constructed the clinical prognostic model by calculating cell senescence-associated prognostic risk scores for each sample. After the screening process of the key genes, we finally identified eight genes related to cell senescence that have clinical significance: IGFBP6, HIST3H2A, GPRASP1, GOLGA8A, COL9A3, CLEC5A, BST2, ATF7IP. In TCGA- GBM cohort, the prognosis of patients with high SRS.score deteriorates with the rise of SRS.score. Conducting the same method on two CGGA cohorts proves the accuracy and stability of our model’s prediction ability.

Subsequently, we found that patients in the high-SRS.score group were older and more male, and there was no mutation in IDH. Interestingly, SRS.score was lower in the C4 and highest in the C1, consistent with the previous survival curve for the C1. Within subgroups of different clinical variables, patients in the high-SRS.score group all had worse prognosis outcomes, which indicates that our risk subgroup had good prediction effect. GBM patients with high SRS.score had poor prognosis and were accompanied by higher immune infiltration.

SRS.score and age showed a significant positive correlation, indicating that the increase in age was also a risk factor for patients with GBM. We also found that the pathways TNFA signaling *via* NFKB, IL2-STAT5 signaling and p53 pathway also showed a significant positive correlation with SRS.score, suggesting that these pathways might be a potential mechanism for poor prognosis in patients with high-SRS.score group. The transcription levels of most immune checkpoints were higher in the high-SRS.score group, and the TIDE score also had a positive correlation with SRS.score, suggesting that the high-SRS.score group was more sensitive to immunotherapy. In chemosensitivity studies, the high-SRS.score group was more sensitive to PD-0332911, just like C1. PD-0332991 is a highly specific inhibitor of cyclin-dependent kinase 4/6 that highly specifically induces G1 phase arrest and thereby inhibits tumor growth (Borghesan et al., 2019). In the high SRS.score group, previously identified genes related to cell senescence characteristics such as IGF-BP6 (Fu et al., 2013) and others significantly increased in transcription level with the increase of SRS.score, and they had the function of promoting cell cycle. The use of PD-0332991 could significantly induce cell cycle arrest, thus playing a role in the treatment of patients with GBM.

Finally, we constructed a decision tree using machine learning and found that SRS.score and age play key factors in the prognosis of patients with GBM. The construction of four risk subgroups based on these two key factors reveals that older patients with high-SRS.score had the worst prognosis, and the C4 subgroup had the highest proportion in the lowest risk subgroup and the lowest proportion in the HIGH group. Subsequent univariate/multivariate COX regression suggested that age, IDH.Mutation, MGMT.promoter.methylation (Bell et al., 2018) and SRS.score were all significant risk factors in the risk factor subgroup.

However, there are some limitations to this study. First, we were unable to demonstrate the differences and roles of disease stages in the progression of GBM due to the lack of information on the GBM progression (such as the tumor stage). Second, our results were derived from bioinformatic analyses and were not further validated experimentally and clinical analysis.

## Conclusion

Molecular subtypes of TCGA-GBM by enrichment scores of seven prognostic cell senescence-related pathways revealed that C1 had the worst prognosis, and at the same time, C1 had the most IDH WT, a higher level of immune infiltration, high immune escape, and downregulation of cell senescence-related pathways, which might be the reasons for C1’ s poor prognosis. We selected eight genes related to cell senescence from TCGA-GBM, which filled the gap in the absence of biomarkers in the cell senescence pathway of GBM.

SRS.score model is robust and independent of clinical pathological features, and has stable prediction performance on independent data sets. Above all, we combined SRS.score with clinical pathological features and adopted decision tree model to further improve the prognosis model and survival prediction, which has high prediction accuracy and survival prediction ability. This provides a basis for the adjuvant treatment of patients with GBM and personalized precision medicine treatment.

## Data Availability

The original contributions presented in the study are included in the article/Supplementary Material, further inquiries can be directed to the corresponding author.

## Supplementary material

Supplementary Figure S1 The flowchart.
