# Supplementary figures and images for: Establishment of a cell senescence related prognostic model for predicting prognosis in glioblastoma
Source: Front Pharmacol. 2022 Dec 6;13:1034794. doi: 10.3389/fphar.2022.1034794 (PMC9763285; doi:10.3389/fphar.2022.1034794)

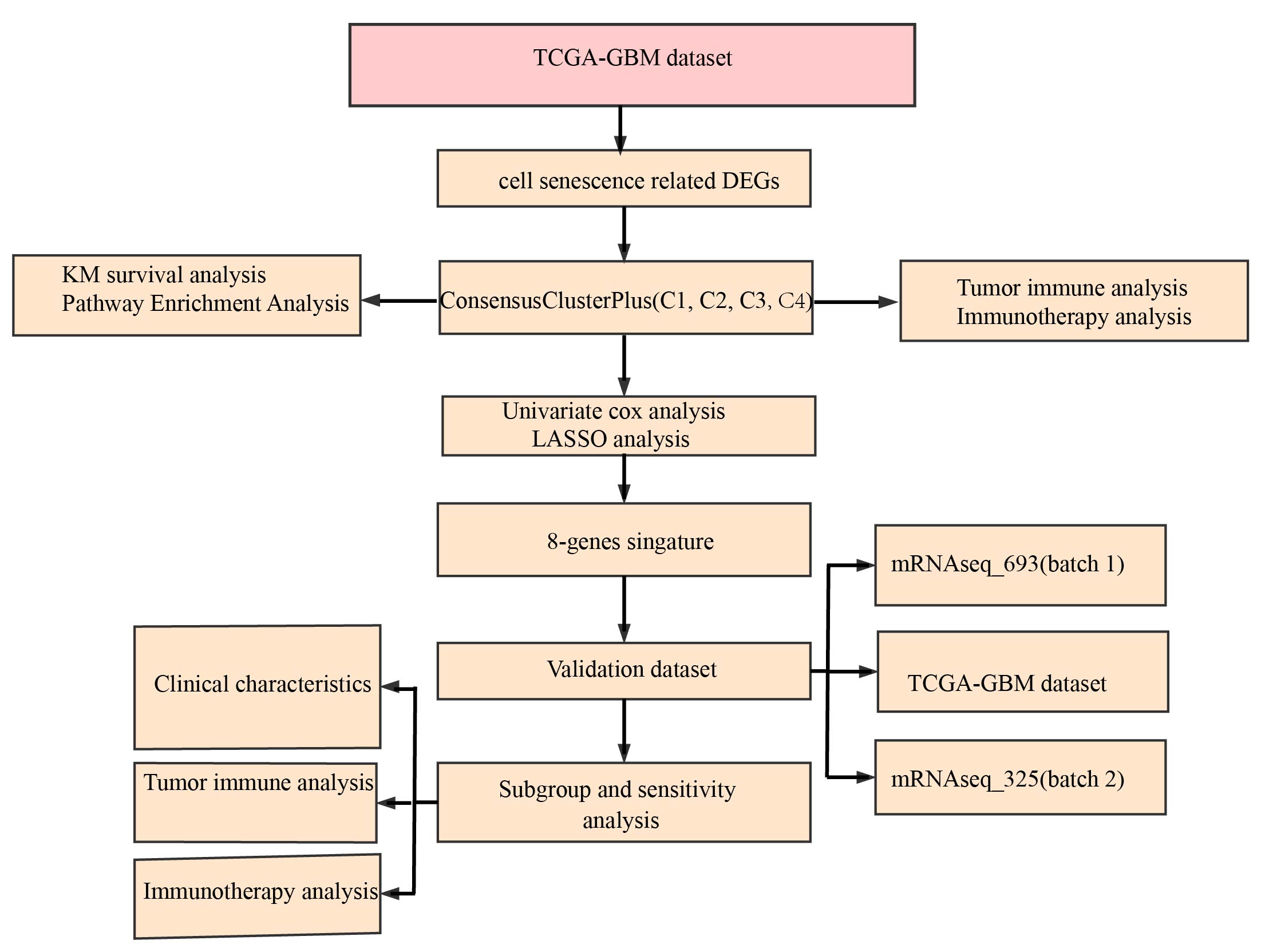

Supplement: Supplementary file 1 [file Image1.JPEG]
